# Computational enzyme design approaches with significant biological outcomes: progress and challenges

**DOI:** 10.5936/csbj.201209007

**Published:** 2012-10-17

**Authors:** Xiaoman Li, Ziding Zhang, Jiangning Song

**Affiliations:** a National Engineering Laboratory for Industrial Enzymes and Key Laboratory of Systems Microbial Biotechnology, Tianjin Institute of Industrial Biotechnology, Chinese Academy of Sciences, Tianjin, Tianjin 300308, China; b State Key Laboratory of Agrobiotechnology, College of Biological Sciences, China Agricultural University, Beijing 100193, China; c Department of Biochemistry and Molecular Biology and ARC Centre of Excellence in Structural and Functional Microbial Genomics, Monash University, Melbourne, VIC 3800, Australia

## Abstract

Enzymes are powerful biocatalysts, however, so far there is still a large gap between the number of enzyme-based practical applications and that of naturally occurring enzymes. Multiple experimental approaches have been applied to generate nearly all possible mutations of target enzymes, allowing the identification of desirable variants with improved properties to meet the practical needs. Meanwhile, an increasing number of computational methods have been developed to assist in the modification of enzymes during the past few decades. With the development of bioinformatic algorithms, computational approaches are now able to provide more precise guidance for enzyme engineering and make it more efficient and less laborious. In this review, we summarize the recent advances of method development with significant biological outcomes to provide important insights into successful computational protein designs. We also discuss the limitations and challenges of existing methods and the future directions that should improve them.

## Introduction

Numerous enzymes have been widely used in biotechnology, pharmaceutical and industrial processes. As biocatalysts are able to accelerate the reaction speed by a factor up to 10^17^ even in mild environments [1], researchers are keen to make certain enzymes applicable in academic, industrial and commercial fields, which has resulted in rapid progress of enzyme engineering in recent years. In particular, great efforts have been made to improve the activity, stability and substrate specificity of the enzymes and design novel catalytic activity. In order to facilitate the modification of target enzymes, a variety of methodologies have been developed. They can be roughly divided into two contrasting categories: rational design and directed evolution [2].

Rational design, the earliest approach applied to the modification of enzymes [3–5], requires the availability of detailed structural information and catalytic mechanism of the targets. Computational tools have been developed to deal with a large number of data produced in rational enzyme design. In the meanwhile, such development leads to the emergence of “*de novo* computational design” approach [6], which commonly refers to the generation of novel protein scaffolds or enzymatic activity. Limited but exciting goals have been achieved in this field [7–9], making *de novo* computational design a promising approach in enzyme engineering. As another common methodology, directed evolution, was only applied to improve desired properties of enzymes recently [10, 11], but it has quickly become a powerful and popular tool in enzyme engineering [12]. Nevertheless, the bottleneck of directed evolution lies in the development of an efficient high-throughput screening technology, despite that there are quite a few successful examples that used directed evolution to modify important commercial enzymes [13–16]. Consequently, the combined approaches involving rational or *de novo* design with directed evolution may offer significant advantages over individual approaches [8, 17].

**Figure 1:**
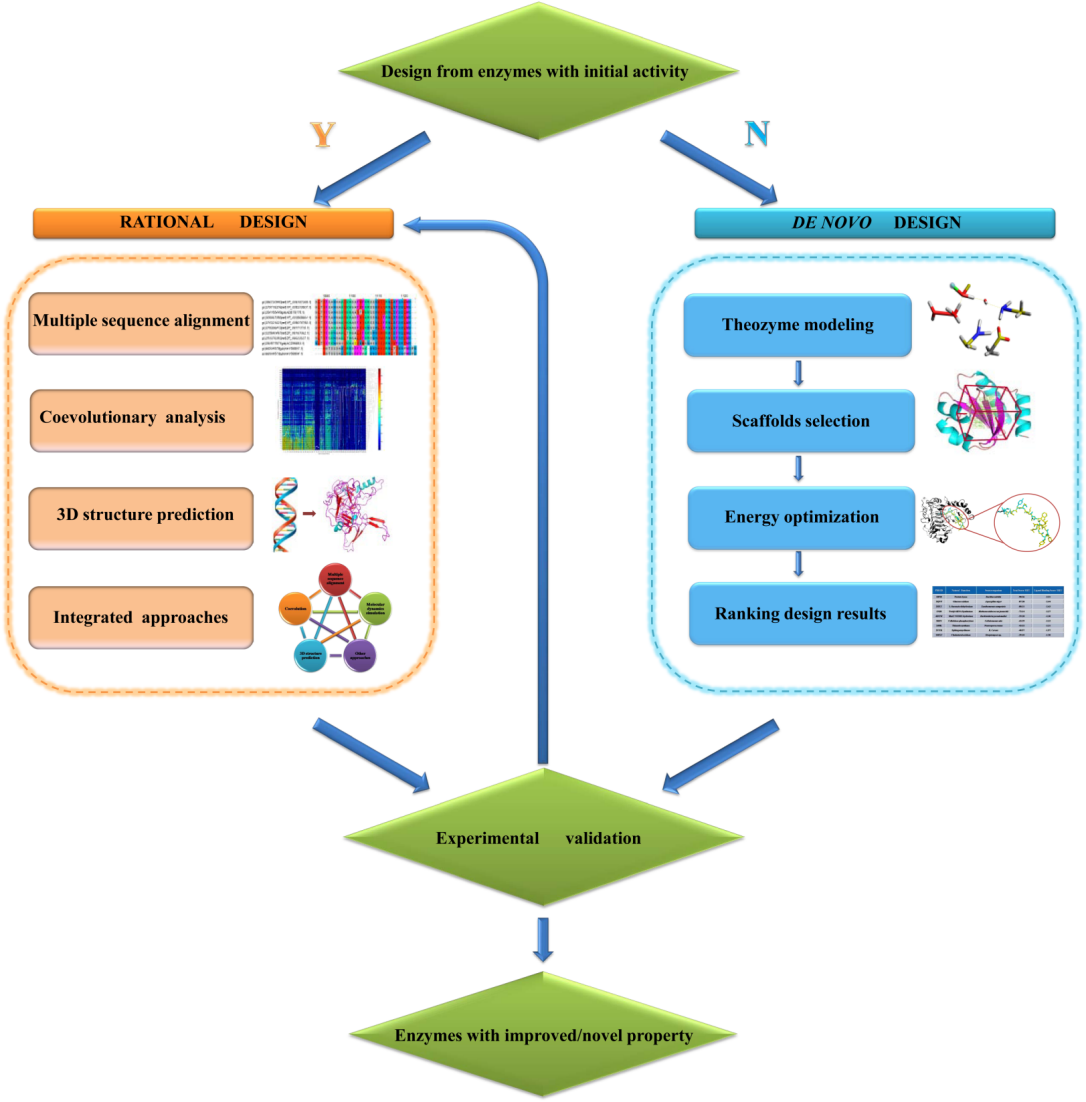
Strategies of rational and *de novo* enzyme design

In this mini-review, we highlight the strengths of a number of effective computational methodologies/tools that can assist in the rational and *de novo* enzyme design (see Figure 1). Successful examples, especially those concerning improvement of enzymatic activity and stability, which are the most important properties from a practical perspective, are discussed in the following respective sections.

## Rational design strategies and tools

The success of rational design depends on our in-depth knowledge about sequence and structure features of target proteins. A popular strategy to identify functionally related residues of unknown targets is the use of sequence features. Analysis of these features can provide enough information about evolutionary relationship, functional sites, correlated mutations and so on. The most useful tools for extracting sequence information are multiple sequence alignment (MSA) and coevolutionary analysis [18], while the latter sometimes requires structural information. As a matter of fact, structure-based design is no doubt more efficient to locate key residues, because the execution of the protein function is directly linked with the maintenance of the 3D structure in functionally related regions. Structure-based rational design can benefit considerably from the rapidly growing number of solved protein structures, however, these account for only a small portion of naturally occurring proteins. To make a better use of structural information, 3D structure prediction or analysis tools are extremely important and greatly desired. Fortunately, a variety of computational methodologies/tools have been available to facilitate processing and data analysis, which have significantly contributed to the progress of rational enzyme design. Among them, several noteworthy tools are discussed below.

### Multiple sequence alignment (MSA)

Protein primary sequence provides the most direct and readily available information for rational design, because important clues for potential mutation sites can be extracted from the amino acid sequence in cases where structural information is not available. For example, Ni *et al*. investigated the activity-related mutations in the wild type of endo-b-1,4-glucanase (RsEG) of *Reticulitermes speratus* via sequence comparison with other cellulases from different sources [19], as well as a RsEG mutant obtained from directed evolution. As a result, they obtained a higher activity and higher expression level of the RsEG mutant. Their analysis identified three single mutants that contributed to a higher enzyme activity, and four residues predicted to be located in the catalytic center by MSA analysis were also experimentally verified. In fact, sequence comparison tends to be more reliable when a reasonable number of homologous sequences are available. High-throughput sequencing techniques have produced larger amounts of data than before. To deal with such data, a variety of MSA methods have been developed in the past two decades [20–22] and have a wide range of applications in modern molecular biology. For rational enzyme design, the construction and analysis of MSA are usually required in the identification of functional-related residues, specificity-determining positions, homology modelling and protein function prediction [22].

Using progressive alignment algorithm [23], a classical MSA method called ClustalW has been widely exploited in various research fields [24–26], and it can generally yield a better performance for highly homologous sequences [27]. For instance, Ehren and co-workers used ClustalW to construct an MSA of 100 homologues of prolyl endopeptidase (PEP) from *Sphingomonas capsulate*, and proposed a list of 30 potentially beneficial mutations based on the generated MSA [28]. A mutagenesis library with limited members was then established, facilitating the selection step and in-depth investigation of each variant. After two rounds of mutagenesis, mutants with enhanced activity and significantly raised resistance to pepsin digestion were identified. In another application of ClustalW, Gumpena *et al*. investigated different proteins from the same gluzincin family. They found that salt bridges that execute similar functions were formed by different residue pairs, and that these salt bridges were not interchangeable, indicating divergent microenvironments around active sites [29]. At present, both ClustalW and its new version ClustalOmega whose accuracy is not influenced by the size of sequences [30], are freely available to the community.

In addition to ClustalW, there are also alternative MSA tools, such as T-Coffee[31], Mafft[32] and Muscle [33], which offer a significantly improved alignment quality with, in some cases, reduced CPU time [34]. Among these, Mafft has been found to be able to provide a consistently better performance in terms of the calculation speed, high quality score with high-throughput data, and high accuracy with very divergent blocks, when evaluated on different benchmarks [35, 36]. Mafft explores two novel methods to enhance its accuracy and scalability [32], which include a fast Fourier transform algorithm that allows rapid identification of homologous regions, and a simplified scoring system designed for CPU time reduction and accuracy improvement of alignments in the case of less homologous sequences. Another iterative refinement technique is also used in Mafft to correct the errors introduced by the progressive alignment [22, 32]. The first version of Mafft was well characterized by a comparable accuracy but shorter CPU time in contrast to ClustalW and T-Coffee, and has been continuously improved in the past ten years [37–39]. The latest version of Mafft is 6.903, which can be run on Mac OS X, Linux, and Windows. Regarding the application of Mafft in protein design, Michel *et al*. compared members of the polysaccharide lyase family 6 with the chondroitin B lyase from *Pedobacter heparinus* [40]. Conserved residues that interact with Ca^2+^ ion were located precisely from the primary sequence, confirming that the chondroitin B lyase has a calcium-dependent catalytic mechanism. MSA analysis was also validated by the X-ray structure and site-directed mutagenesis. In the follow-up enzyme engineering step, the redesign of such function-related residues can be avoided in advance. Maita *et al*. also employed Mafft to perform an MSA analysis of oligosaccharyl transferases (OSTs) from different microbial domains [41]. After inclusion of a considerable number of distantly related sequences, Mafft yielded a satisfying performance and facilitated the identification of three different kinds of catalytic centers. Furthermore, they also found that two distantly related OSTs share a higher structural similarity than sequence similarity. These results indicate that the application of additional information in MSAs, such as sequence homologs and structural information, can improve the MSA quality [20].

In addition to the improvement of computational algorithms, there is another trend that involves a combination of several MSA methods based on the same set of sequences. The work on 3-deoxy-D-manno-octulosonate 8-phosphatesynthases (KDO8PS) by Ackerman *et al*. provided a good example [42]. In that work, Mafft, T-Coffee and Muscle programs were used individually for curating the MSAs of all known KDO8PS, with the results further integrated using T-Coffee. Seven pairs of coevolved residues were identified, and their contribution to protein stability was examined. Interestingly, one mutation in one coevolving residue pair that resulted in a slight decrease in protein stability could be compensated by another mutation in the same pair to maximize the stability of the protein. These results highlight that an important property, “coevolution”, extracted from a curated MSA of protein sequences, can provide a meaningful research direction for rational enzyme design.

### Coevolutionary analysis

Coevolution (also known as covariation, correlated mutation or co-substitution) refers to “reciprocal evolutionary change in evolutionarily interacting loci” [43], and occurs at many levels in biology [44–46]. In this review, only the correlated mutations between amino acids within a protein are discussed. Coevolutionary analysis methods have a number of important applications in the prediction of protein structure [47, 48], identification of functional sites [49–51] and candidate design sites [52, 53]. The identified coevolving residues have been experimentally validated in some studies [54, 55], implying the potential application of coevolutionary analysis in rational enzyme design.

In the past few decades, a number of coevolutionary analysis algorithms have been developed [56]. These methods share a common procedure of three steps: MSA construction, coevolutionary measure calculation and experimental validation. Most coevolutionary analyses start with the construction of an MSA of the query protein. Although certain automatic software can be applied (see Table 1), manual refinement, including filtering of sequences with large gaps, low homology or wrong annotation, is often required to ensure a high-quality MSA [57]. The second step is to calculate coevolutionary measures, which can be done by using different correlated mutation algorithms, followed by statistical significance tests and analyses to extract significant coevolution values, eliminate background noise [58] and evaluate the performance and robustness of the coevolution measures [59]. Finally, “wet” experiments need to be performed to validate the obtained coevolutionary results.

**Table 1:** Summary of useful computational programs in rational design referred in this review.

Programs	Application	URL address	Operating system	Ref.

Rational design programs				
ClustalW		http://www.clustal.org/clustal2/	Windows, Linux, MacOS	[27, 122]
ClustalOmega		http://www.clustal.org/omega/	Windows, Linux, MacOS	[30]
Mafft	Multiple sequence alignment	http://mafft.cbrc.jp/alignment/software/	Windows, Linux, MacOS	[32, 37] [39]
T-Coffee		http://www.tcoffee.org/Projects/tcoffee/	Linux, MacOS	[123]
Muscle		http://www.drive5.com/muscle/	Windows, Linux, MacOS	[33]
Integrated system		http://coevolution.gersteinlab.org/coevolution/	Windows, Linux, MacOS	[60]
OMES-KASS	Coevolutionary analysis	http://bip.weizmann.ac.il/correlated_mutations/	Linux	[63]
Fodor package		http://www.afodor.net/	Windows, Linux, MacOS	[61]
Swiss-Model		http://swissmodel.expasy.org/	-	[124, 125]
HHpred2	3D structure prediction	http://toolkit.tuebingen.mpg.de/hhpred	-	[83]
I-TASSER		http://zhanglab.ccmb.med.umich.edu/I-TASSER/	Linux	[84, 126]
FoldX		http://foldx.crg.es/	Windows, Linux, MacOS	[96, 127]
PopMuSiC	Protein stability prediction	http://babylone.ulb.ac.be/popmusic	-	[94, 97] [128]
I-Mutant3.0		http://gpcr2.biocomp.unibo.it/cgi/predictors/I-Mutant3.0/I-Mutant3.0.cgi	-	[129]
DMutant		http://sparks.informatics.iupui.edu/hzhou/mutation.html	-	[130]

***De novo* design programs**				

RosettaMatch	Scaffold search	-	-	[108]
RosettaDesign	Protein design for low free energy sequences	http://rosettadesign.med.unc.edu/	Linux	[109]
ORBIT	Optimal sequences search for given folds	-	-	[118]

For experimental scientists, coevolutionary webservers seem to be more straightforward, attractive and practical. Up to now, several online tools have been made publicly available [56, 60]. However, how to choose an optimal scoring function of coevolutionary measures in the second step remains to be a critical factor that will determine the quality of coevolutionary analysis. To address this, Fodor *et al*. [61] assessed the performance of four different methods in detecting coevolutionary site, namely Statistical Coupling Analysis (SCA) [62], Observed Minus Expected Squared (OMES) [63], McLachlan Based Substitution correlation (McBASC) [64] and Mutual Information (MI) [57]. In their research, OMES and McBASC were found to outperform the other two algorithms in favoring poorly conserved residue pairs and decreasing sensitivity to background conservation, and were of considerable similarity in sensitivity to background noise. The OMES-based programs, OMES-KASS [63] and Fodor package [61], which were more recently developed, have been applied to perform reliable coevolutionary analysis [65–67]. In addition, Yip *et al*. developed an integrated online program by embedding several coevolutionary algorithms into one system instead of using a single algorithm only. These algorithms include SCA, MI, Explicit Likelihood of Subset Variation (ELSC) [68] and correlation-based methods [64, 69], making this system a convenient comparative analysis tool of different coevolutionary methods. The integrated system also provides an MSA preprocessing option to further improve its performance. In addition, users can also choose to treat the gaps in the MSA as noise or as an additional 21st residue, based on the observation that gaps might contain important coevolutionary information [60].

Despite the functional significance, how to combine coevolutionary analysis with rational enzyme design remains a challenging issue. In 2011, Zeng and colleagues applied SCA to analyse the sequences of the regulatory domains of the aspartokinase (AK) family to characterize the allosteric interaction network [53] and integrated such information with rational enzyme design. AK is the central enzyme in the biosynthesis of aspartate family amino acids, and the allosteric inhibition of AK by end-products obstructs the production of related amino acids in *Corynebacterium glutamicum* [70]. As a result, their coevolutionary analysis of 500 sequences from the AK family identified 25 highly correlated positions, in which 14 sites were mutated to construct AK mutants of *C.glutamicum*. All the mutants showed resistance to allosteric inhibition to different extents, suggesting that the choice of target mutations was largely successful. In this study, a major strategy was to select residues that had the potential to interrupt allosteric interaction, whereas in researches that aim to modify other properties of enzymes, amino acidsites that regulate the target property can probably be selected as candidates according to expert knowledge or structural analysis. There were two general rules to mutate the wild-type amino acids at the selected sites: (i) mutating the wild-type amino acids to those with less usage frequency at the corresponding positions; (ii) or substituting the wild-type amino acids by those with different chemical properties with the purpose of making more obvious changes in terms of the target properties [53]. In another work of Chen and co-workers, AK3 from *Escherichia coli* was investigated via an integrative analysis of coevolution and molecular dynamics (MD) [71]. The SCA-based coevolutionary analysis of 340 protein sequences with 424 positions was combined with the 10 nanosecond (ns) MD simulation of AK3 with/without lysine as an effector molecule. 30 top ranked positions were accordingly selected, most of which were reported as potential targets for point mutations in other studies using random mutagenesis. The site-directed mutations of the remaining positions not found by random mutagenesis, however, led to significant deregulation of allosteric inhibition by effectors. Although both coevolutionary analysis and MD simulation are complicated, usually requiring iterative procedures prior to the result generation, they have better efficiencies than traditional experimental approaches like random mutagenesis. In the case of AK3, its computational design can be “grafted” into another AK of the same family even with lower sequence identity, making it more efficient and appealing.

### Protein 3D structure prediction

There are an increasing number of proteins with high-resolution solved 3D structures, greatly facilitating the rational and computational protein design. Numerous previous successes have shown that when 3D structural information is available, protein design can be much more precise and accurate [18, 72, 73]. It is apparent that the knowledge of 3D structure of the target enzyme is a prerequisite and foundation for structure-based design. Although only a small portion of proteins have authentic crystal structures, those with unknown structure information can be reliably modeled via protein 3D structure prediction software, provided that there is a known structure of one or several homologous proteins to the target protein [74, 75].

According to the availability of template structures, protein 3D structure prediction can be generally divided into two categories: homology modelling and *ab initio* modelling. The former refers to the construction of an atomic-resolution model of a protein from its primary sequence using the experimentally solved 3D structure of a homologous protein as the “template”, while the latter is called “free modelling” or “*de novo* modelling” in some cases, referring to 3D structure prediction generated from scratch when structural analogs are not available or detectable. The majority of methods used in homology modelling can be further grouped into two types: comparative modelling (CM) [76] and threading [77]. The root mean square deviation (rmsd) of a CM constructed model from the structure obtained from experiments can usually achieve 1–2 Å when a highly homologous (>30% sequence identity) template is employed. Models with such accuracy can compete with the low-resolution X-ray or medium-resolution NMR structures [78]. In contrast, the threading approach usually has a remarkable performance when dealing with target protein modelling using relatively distant templates, and the corresponding rmsd is 2-6Å [79] with most errors occurring in loops. *Ab initio* modelling, however, continues to be the most challenging topic in protein 3D structure prediction. Although there has been an exciting progress in modelling small proteins, no substantial progress has been achieved in *de novo* structure prediction of proteins with more than 150 residues [80]. In view of this, we mainly focus on the homology modelling methods in this mini-review.

According to the initial plan of protein Structure Initiative (PSI), proteins within 90% of the domain families can be modeled by CM at its completeness [81]. As a consequence of this project, homology modelling is becoming increasingly important. Nowadays, a handful of academic-free servers for template-based protein structure prediction are available without any restrictions, resulting in a confusion about which tool should be used for solving different tasks. A popular criterion to assess the 3D structure prediction quality is the Critical Assessment of Structure Prediction (CASP) which has been carried out each two years since 1994 [76]. In the latest competition, CASP9 in 2010 [82], 176 groups took part in the homology modeling which is the most relevant category for biological applications. According to the results of the assessment, a group of six methods have outperformed noticeably the rest ones in the “server” category [82], among which HHpreB [83] and Zhang-Server (namely I-TASSER) [84, 85] were assessed as the best.

However, no matter how significantly an algorithm has been improved, the modelling quality greatly relies on the sequence homology between the template and the target. The prediction procedure can be further simplified and become straightforward when a closely related template is available. Besides, meta-server, which produces a combined prediction using results of other automatic servers, has proved to outperform most individual ones [86]. Due to page limitation, only the popular automated webservers that suit protein design purposes are reviewed in this section. Swiss-Model [87], an automated CM server, is regarded as the most widely used online tool in protein 3D structure prediction. CM, as described above, is the only methodology that can reliably model a 3D structure using amino acid sequence alone [88]. By submitting an amino acid sequence or its UniProtID, users start the modelling procedure with or without providing a template protein. Swiss-Model server can automatically select several suitable templates from a refined library derived from the Protein Data Bank (PDB), and then a structural alignment between the target and the template is generated and improved for the sake of modelling [87]. The mapping of the residue correspondence between the target and the template begins at this step, followed by model building. In the Swiss-Model server, three building modes can be selected before the submission: “automated”, “alignment” and “project”; it is recommended to choose options according to the similarity between the target and templates [89]. “Automated” is for higher similarity of >60%, “project” for that below 20%, and “alignment” otherwise. The energy minimization of the built models by the GROMOS96 force field is the final step. Efforts have been made to improve the modelling quality of Swiss-Model since it was developed. Numerous examples have been provided in literature, and some representatives are discussed here. The Kir2 channels are a kind of potassium selective channels [90]. A pH sensitive member Kir2.3 was aligned with all the Kir2 channel proteins, and histidine 117 (H117) located close to the putative selectivity filter was identified to contribute to pH sensitive phenotype [91]. However, contradictory results were obtained by directed mutagenesis experiments, suggesting that there were other factors related to the pH effect. The observation that the ability of Zn^2+^ to bind cysteines/histidines could inhibit the pH effect indicated that a cysteine within atomic distance to H117 might interact to exert this functional effect. Consequently, the 3D structure of Kir2.3 was created by Swiss-Model using distant templates in order to narrow down the range and locate the target cysteine. The rational design of candidate sites was implemented by site-directed mutagenesis, and C141 was found to interact with H117 to exert an influence on pH sensitivity. In another example, Choi and colleagues carried out homology modelling-based rational design of an epoxide hydrolase (EH) in a marine fish, *Mugil cephalus* [92]. The 3D structure of EH from a fungus, *Aspergillus niger*, was selected as the template by Swiss-Model for 3D structure prediction of *M.cephalus* EH. The active sites of the predicted structure were then superimposed on the template and indicated that the spatial orientation of D199 in the target EH was different from its counterpart in the template. Attempts to modify D199 into a proper orientation were also made to redesign the surrounding residues so that they could have direct or indirect interactions with D199. To achieve this, F193 and Y194 were chosen, and the 3D structures of various mutants of these two residues were constructed by Swiss-Model instead of “wet” experiments. Analysis of the corresponding 3D structures, particularly the activity sites, revealed that D199 had the right orientation in the variants F193Y and Y194M. Site-specific mutagenesis confirmed that the F193Y mutant indeed improved the catalytic activity and decreased the reaction time. It is worth noting that the reliability of Swiss-Model prediction was validated in a situation where a distant template was used, providing a good example of freeing researchers from laborious experiments by entirely resorting to the Swiss-Model computational tool.

A closely related issue is protein stability design. In a recent work on glycerol dehydratase (GDHt) [93], prediction of protein stability was realized by a computational program called PoPMuSiC [94]. The selection of point mutation residues mainly depended on the prediction result. The performance of such tool requires the 3D structure of the target protein. Accordingly, homology modelling of the target GDHt was first conducted by the Swiss-Model server based on the template retrieved from PDB (ID: 1IWP). Two mutations that were predicted to be the most stable were selected and mutated by single point mutation. The 3D models of the two mutants were built again using Swiss-Model. An enhanced hydrogen bond interaction between the mutated positions and the surrounding residues accounted for the improved stability, which was validated by experiments. We conclude from a large number of examples including those discussed above that 3D structure prediction provides not only direct evidence for rational protein design, but also essential assistance for structure-based enzyme redesign. Since less than 1% proteins have solved 3D structures, studies on the stability and other important properties of most target proteins have to rely on the predicted structural information.

Unfortunately, there are no generally applicable rules for enzyme activity enhancement, due to the variance in catalytic mechanisms of different types of enzymes. Therefore, many efforts have also been made to improve other important properties of enzyme catalysts, for example, protein stability, a critical property of an enzyme catalyst that is pertinent to its industrial potential. As Swiss-Model and many other predictors can produce high-quality results, a crucial step in protein stability prediction is the choice of well-performing servers. According to a recent systematic analysis of 11 online stability predictors by Khan and Vihinen [95], FoldX [96] is amongst the top ones. However, FoldX does not provide a convenient online webserver, which has limited its broad application. Another well-performing tool PoPMuSiC provides an alternative choice, which was developed in 2000 [97] and updated in 2009 [98] using more experimental data from ProTherm [99]. The most-recent version of PoPMuSiC webserver was released in 2011 [94], providing a systematic evaluation on stability changes under saturated single-site mutations at each residue position, or an appointed one for the submitted protein on the basis of its 3D structure.

### *De novo* computational design

The ultimate test of our understanding of the mechanism of enzymatic catalysis is *de novo* computational design, which refers to creation of novel protein folds, substrate binding pockets, and catalytic activities and so on. *De novo* protein design was first conducted to create a four-helix bundle protein in 1988 [6]. Since then, various protein folds have been *de novo* designed [100]. However, only a few possessed catalytic functions. Accordingly, *de novo* computational design of naturally occurring enzymes with novel catalytic activity is considered as a grand challenge, and in recent years, great efforts in this field have been made to expand our knowledge in enzyme engineering [7–9, 101–103]. To illustrate this, in this section we discuss three distinguished design examples of enzymes that catalyze synthetic reactions.

The overwhelming performance of enzymatic catalysis over chemical catalysis is partly due to the free energy decrease of transition state (TS) via the interaction with catalytic residues [104]. Hence, the first step of *de novo* design for a given reaction is to model its theozyme which consists of TS model and catalytic groups [105] based on quantum chemical calculations [106]. How well the theozyme models correlate with their corresponding crystal structures, will have a significant influence on the ultimate designs. Dechancie *et al*. mimicked the active sites of nine distinct enzymes with quantum mechanical optimizations [107]. The rmsd of the sets of catalytic atoms was 0.64Å, suggesting that the predicted geometries were remarkably consistent with the corresponding X-ray structure. For a desired reaction, there usually exist more than one possible catalytic mechanism. As result, the 3D models of each catalytic motif for each mechanism will have to be built, and hence the degree of freedom and the orientation of different bonds in each model can vary greatly, giving rise to a great number of possible 3D active sites, which are called “theozyme library”.

The search for optimal protein scaffolds that are able to fulfill a target reaction can be launched once the theozyme library has been generated. Numerous scaffolds with ligand-binding cavities and high-resolution X-ray structures are available in several public protein databases. If there are certain restrictions on potential scaffolds, for example, in cases where a thermophilic scaffold is required, the selection range could be narrowed down. However, this process depends on *de novo* design algorithms such as RosettaMatch [108] that relies on hashing techniques and pruning of the majority of potential active centers at a very high speed but very little cost. At this step, the description of TS and a set of protein scaffolds are input into RosettaMatch. Once a TS position is compatible with the geometry of catalytic sites in one scaffold and satisfies other catalytic constraints, the result will be output as a “match” [106, 108].

Because there are still substantial candidate matches after the scaffold selection, and there remain certain steric clashes between the TS position and the catalytic side chains in the matches, further optimization is necessary. In this regard, the RosettaDesign methodology [109] can be applied to improve the binding affinity to TS and the stability of the active centers by redesigning or repacking of related residues. It is suggested that users run a single task for ten times owing to astochastic sampling algorithm adopted by RosettaDesign which will probably produce 10 distinct outputs. The resulting designs are supposed to be lower energy sequences for a given scaffold with the maximized TS affinity.

After optimizing all unique matches, a next step is to select designs with optimal performance for experimental validation. Several important factors, especially the ligand binding energy feature, are often used to evaluate and rank all the designs as described in [106]. As it is unlikely that a design has the highest score for each factor, extensive examinations to assist in further selection are preferred. In addition, Kiss *et al*. found that the MD technology was the most effective procedure for predicting the catalytic potentiality of designs [110].

The same protein scaffolds can execute diverse functions, such as α/β–barrel motif, which constitutes approximately 10% of proteins that perform a wide range of catalytic reactions [111]. This indicates that the designable potentiality of certain scaffolds underlies the foundation of computational engineering of novel functions. With similar strategies, Baker's group has performed a series of pioneering studies in redesigning enzymes that catalyze retro-aldol reaction [7], Kemp elimination [8] and Diels-Alder reaction [9].The enhancement of target reactions by designed enzymes was assessed by the ratio of the catalytic rate constant and uncatalyzed rate constant *k*_cat_/*k*_uncat_. In the above cases, the values of *k*_cat_/*k*_uncat_ ranged from 10^2^ to10^5^ for the most active designs, indicating the effectiveness of such design strategies. *De novo* computational enzyme design provides important insights into the structure-function relationship of the enzyme and the starting points for directed evolution and rational design. Considerable experimental efforts, including development of technologies discussed in the *Rational design strategies and tools* section, were made to enhance the activities of the artificial Kemp eliminases [112–114].

While the *Rosseta*-based *de novo* design is well characterized by its own scaffold selection steps, it is worth noting that other types of *de novo* design approaches are emerging recently and have achieved an impressive success, which were also developed based on a given scaffold [101, 115–117]. Once a suitable protein scaffold is selected according to the desirable properties of the target reaction, such as thermostability, high expression level and presence of cofactor binding domain, *de novo* design approaches only need to build an activity center and a substrate/cofactor binding pocket. In this regard, Bolon and Mayo presented a representative example of a “compute and build” strategy [115]. They chose *E. coli* thioredoxin as the starting scaffold due to its favorable thermostability and expression in *E. coli*, and used the p-nitrophenyl acetate (PNPA) hydrolyzation regulated by a histidine as the target reaction. A computational algorithm called ORBIT (optimization of rotamers by iterative technique) [118], was applied to scan active sites in the starting scaffold. Two catalytic sites were identified, and mutations surrounding these catalytic sites were then introduced in order to build compatible substrate binding pockets. Two resulting designs were further experimentally validated. One design PZD2 reached a *k*_cat_/*k*_uncat_ value of 180. In another example, *de novo* design of a functional metalloprotein, namely the nutric oxide reductase (NOR), was performed by Yeung *et al*. [117]. The goal was to build a non-haem Fe^2+^-binding site (Fe_B_) in the scaffold sperm whale myoglobin (Mb). Based on the structural information of a structural homologue with a haem-copper site, two residues L29 and F43 were mutated to histidines, which constituted the Fe_B_ center together with H64 and V68E. Modelling analysis using an extension of Visual Molecular Dynamics (VMD) was performed to build the designed protein Fe_B_Mb, suggesting the formation of the Fe_B_. Subsequent crystal and experimental data confirmed the accuracy of the predicted model and an apparently increased activity. These examples discussed above highlight the importance and complementarity of these alternative *de novo* design strategies, which can be applied to similar scaffold-based studies.

## Discussions

In this mini-review, we aim to provide a useful guide on the selection of the basic design methodologies and tools that are frequently employed in enzyme engineering (Table 1), and a brief summary of these aforementioned examples is depicted in Table 2. For many naturally occurring enzymes, it is often necessary to modify and design their properties in order to meet the needs of commercial or industrial applications. Bioinformatic strategies and tools, particularly those with freely accessible webservers, offer biologists tremendous help to narrow down their experimental efforts.

**Table 2:** Summary of representative examples referred in this review.

Enzyme/protein	Target property	Method		Result			Ref.

		Design strategy	Bioinformatic tool	No. of mutants	Fold-improvement	Library size	
*R.speratus*endo-β-1,4-glucanase	Activity	Functional and activity-related residues identified via an MSA analysis of eight sequences	-	7	7-13	24	[19]
*S.capsulata*	Activity; stability	An MSA of 100 homologues evaluated by multiple scoring functions identified mutations	ClustalW, SeqDist, KaKs, probCons, SUB	6(1st round)	20%(activity)	47(1st round)	[28]
prolyl endopeptidases				9(2nd round)	200(stability)	48(2nd round)
KDO8P Synthase family	Stability	Integrated analysis by MSA, ΔΔG changes calculation, MD simulation and coevolutionary analysis	Mafft, T-Coffee, Muscle, HMMER 3.0, Prime 2.1, Desmond, X-Cluster, FoldX	No experimental validation			[42]
*C.glutamicum* aspartokinase	Allosteric inhibition	Correlated positions were identified by coevolutionary analysis of 500 sequences	Muscle, ClustalX	1	2	14	[53]
*E.coli* aspartokinase	Allosteric inhibition	Integrated analysis byMD simulation and coevolutionary analysis	Modeller, AMBER, Muscle	6	5-7	18	[71]
*M. cephalus* epoxide hydrolase	Activity	Activity-related residues were identified by superimposition of a predicted structure and a solved structure template	Swiss-Model, RasMol, Deep-View	1	35	5	[92]
*K. pneumonia* glyceroldehydratase	pH stability; activity	Stability-related residues were designed based on a predicted structure	Swiss-Model, PoPMuSiC	1	2(pH stability) 2(activity)	2	[93]
Retro-aldol reaction	Activity	TS simulated by QM/MM was used for scaffold selection and followed by individual optimization and ranking	RosettaMatch, RosettaDesign	32	2 × 10^4^	72	[7]
Kemp elimination				8	>10^6^	59	[8]
Diels-Alder reaction				2	89M	84	[9]
*E. coli*	PNPA	Potential active sites and surrounding active-site mutations were identified and computed	ORBIT	2	180	2	[115]
thioredoxin	hydrolase						
Sperm whale myoglobin	Nitric oxide reductase	Creating a non-haem Fe^2+^-binding site based on the predicted structure overlaid with the reference structure	VMD, NAMD	1	N.A.	<10	[117]

N.A.: Not Available

MSA can efficiently identify consensus, highly conserved and variable positions within a family of homologous proteins, while MSA-based coevolutionary analysis of a set of enzymes with similar functions provide critical clues about catalytic and other functionally related residues. A number of candidate sites derived from these sequence-based studies can be used to construct a mutation library, facilitating the discovery of favorable mutants with improved functional properties. On the other hand, with the increasing availability of high-quality 3D structures in the PDB, there are a growing number of structure-based approaches being developed. Because experimentally solved structures only cover a limited portion of the protein repertoire, sequence-based 3D structure prediction has become a prevalent methodology in enzyme engineering. This is important, because reliable prediction of protein structure can still provide valuable information regarding potential candidate sites whose mutations might lead to improved properties of the enzyme, even if its real structural information is not at hand. As a symbol of the engineering of the third wave of biocatalysts [119], *de novo* enzyme design has achieved a significant success in the last 20 years. Despite these advances, there are challenges for rational enzyme design. A first challenge is that there are inevitable experimental errors in “wet” experiments [120], resulting in less reliable designs based on such low-quality data. A second challenge is related to the conformational dynamic nature of the enzyme. Conformational changes of the enzyme are frequently occurring under catalytic conditions, leading to a deviation of the real orientation of residues and enzyme structure from that of the designed or modeled enzymes. A third challenge is how to select the most appropriate tool that best suits the study of a particular target enzyme, from a pool of different tools that have both pros and cons. In this mini-review, we attempt to provide a useful guide to summarize some of the popular, reliable and academic free tools. Moreover, many examples have proved that integrative strategies can usually outperform individuals. In this regard, development of meta-servers is promising for providing a better performance and reliability of computation design. A fourth challenge is that some modified catalysts still cannot meet the practical needs of large-scale applications, particularly *de novo* designed enzymes. As such, there is often a need for assistance of experimental approaches, such as directed evolution. In fact, the boundary of rational design and directed evolution has become more and more blurred in practical applications, as evidenced by a number of recent studies that involve a combination of both [5]. Therefore, improving experimental techniques, such as high-quality mutagenesis and high-throughput screening, is another related future direction.

Due to the aforementioned challenges, many attempts of computational protein design failed. However, future development of the field will be advanced by a better understanding of the underlying reasons that led to both failures and successes [121]. Recent advances in computational enzyme design have largely expedited the evolution of enzymes, and have greatly revolutionized the way of enzyme engineering. With the development of improved experimental techniques, computational enzyme design will gain a momentum and achieve significant successes in the future.
